# Role of Cross-Sectional Imaging in Pediatric Interventional Cardiac Catheterization

**DOI:** 10.3390/children9030300

**Published:** 2022-02-22

**Authors:** Yousef Arar, Abhay Divekar, Stephen Clark, Tarique Hussain, Roby Sebastian, Mehar Hoda, Jamie King, Thomas M. Zellers, Surendranath R. Veeram Reddy

**Affiliations:** 1 Department of Pediatrics, University of Texas Southwestern Medical Center, 5323 Harry Hines Blvd, Dallas, TX 75390, USA; abhay.divekar@utsouthwestern.edu (A.D.); stephen.clark@utsouthwestern.edu (S.C.); mohammad.hussain@utsouthwestern.edu (T.H.); roby.sebastian@utsouthwestern.edu (R.S.); mehar.hoda@utsouthwestern.edu (M.H.); thomas.zellers@utsouthwestern.edu (T.M.Z.); suren.reddy@utsouthwestern.edu (S.R.V.R.); 2 Pediatric Cardiology, Children’s Medical Center, 1935 Medical District Dr, Dallas, TX 75235, USA; jamie.king@childrens.com; 3 Department of Radiology, University of Texas Southwestern Medical Center, 5323 Harry Hines Blvd, Dallas, TX 75390, USA; 4 Department of Anesthesia and Pain Management, University of Texas Southwestern Medical Center, 5323 Harry Hines Blvd, Dallas, TX 75390, USA

**Keywords:** congenital heart disease, interventional cardiology, cross sectional imaging, cardiac computed tomography (CT), cardiac magnetic resonance (CMR)

## Abstract

Management of congenital heart disease (CHD) has recently increased utilization of cross-sectional imaging to plan percutaneous interventions. Cardiac computed tomography (CT) and cardiac magnetic resonance (CMR) imaging have become indispensable tools for pre-procedural planning prior to intervention in the pediatric cardiac catheterization lab. In this article, we review several common indications for referral and the impact of cross-sectional imaging on procedural planning, success, and patient surveillance.

## 1. Background

Historically, echocardiography has been the primary imaging tool used to help guide percutaneous interventional procedures in the congenital heart disease (CHD) population. Cross-sectional imaging is now emerging as an essential complement to patient selection and monitoring. With improvement in pre-procedural imaging, catheterization procedures are becoming more directed toward interventions and less diagnostic.

Cardiac computed tomography (CT) and cardiac magnetic resonance imaging (CMR) have made substantial improvements in sequencing, spatial resolution, and reduction in radiation exposure to the point where they are now routinely being employed to assist with pre-procedural planning. Meticulous pre-procedural planning has now led to significantly improved outcomes in the cardiac catheterization laboratory. 

This review article will focus on how cross-sectional imaging aids interventional cardiologists in the catheterization lab. Echocardiographic–fluoroscopic fusion imaging and interventions play a substantial role; however, this topic will be omitted for the purposes of this review article. Case examples will be presented in the general format of pre-procedural, intra-procedural, and post-procedural considerations.

## 2. Case Examples

We have selected specific case examples to illustrate the uses of cross-sectional imaging to aid with percutaneous congenital cardiology interventions.

### 2.1. Sinus Venosus Atrial Septal Defect (SVASD)

SVASD are characterized by an embryological defect between the wall of the superior vena cava (SVC) and right upper pulmonary vein (RUPV), leading to a significant left to right shunt [1,2,3]. Cross-sectional imaging has played a vital role in the diagnosis and interventional planning for percutaneous closure. Not all patients are suitable candidates for transcatheter intervention as many factors must be considered pre-operatively including pulmonary vein insertion, length of SVC, and sites of possible obstruction from the covered stent placement. 

**Figure 1:**
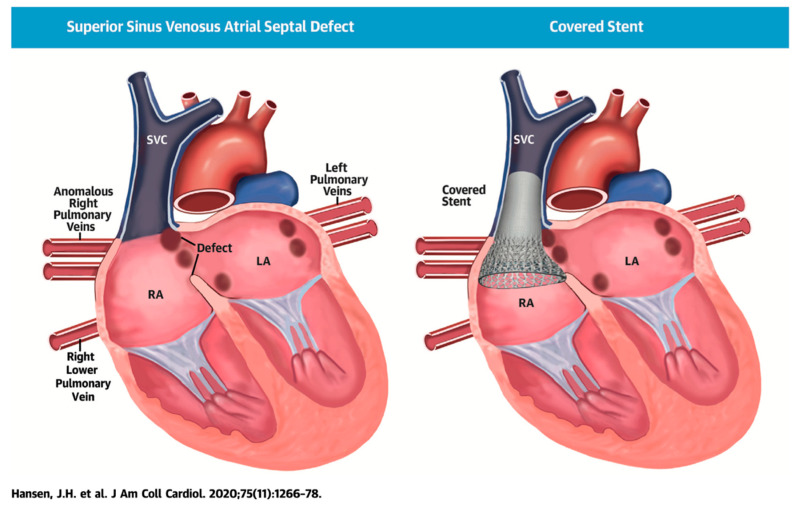
Illustration of successful covered stent placement with closure of the SVASD. The anomalous pulmonary veins now drain directly to the left atrium (LA) while the SVC drains entirely to the RA. Revised from ref [4].

**Figure 2:**
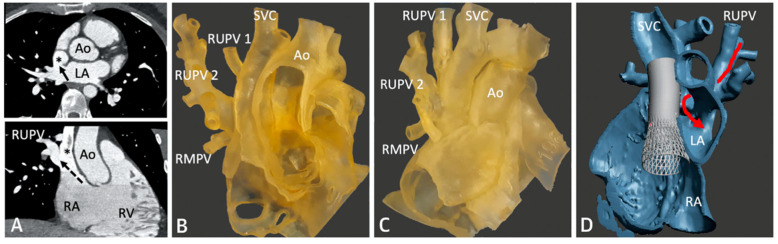
Series of images depicting pre-procedural planning with CT (**A**), three-dimensional (3D) printed models (**B**,**C**), and 3D virtual modeling (**D**). The defect is represented by an asterisk (*) in (**A**). After review with 3D imaging, each patient was either referred for covered stent placement (**B**, **D**) or surgical repair (**C**), if deemed inappropriate for percutaneous closure. Revised from ref [4].

**Figure 3:**
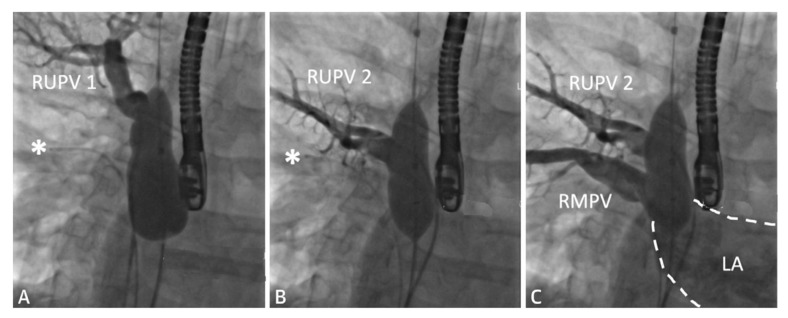
Series of fluoroscopy images depicting balloon test occlusion prior to covered stent placement. The patient was deemed to be an unsuitable candidate for covered stent placement as both RUPVs demonstrated an obstructive pattern returning to the LA (**A**,**B**). Venous return from the RMPV (**C**) was normal. A catheter is seen at the asterisk (*). Revised from ref [4].

**Figure 4:**
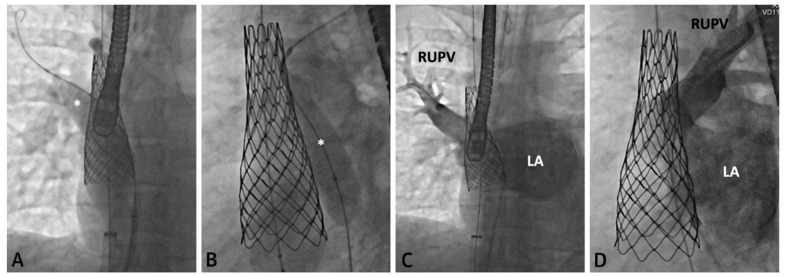
Series of fluoroscopy images depicting stent placement. An Atlas Gold Balloon (*) is placed in the RUPV to prevent obstruction during SVC covered stent deployment (**A**,**B**). Follow up hand injection angiogram in the RUPV demonstrates no residual narrowing and normal venous return (**C**,**D**). Revised from ref [4].

**Figure 5:**
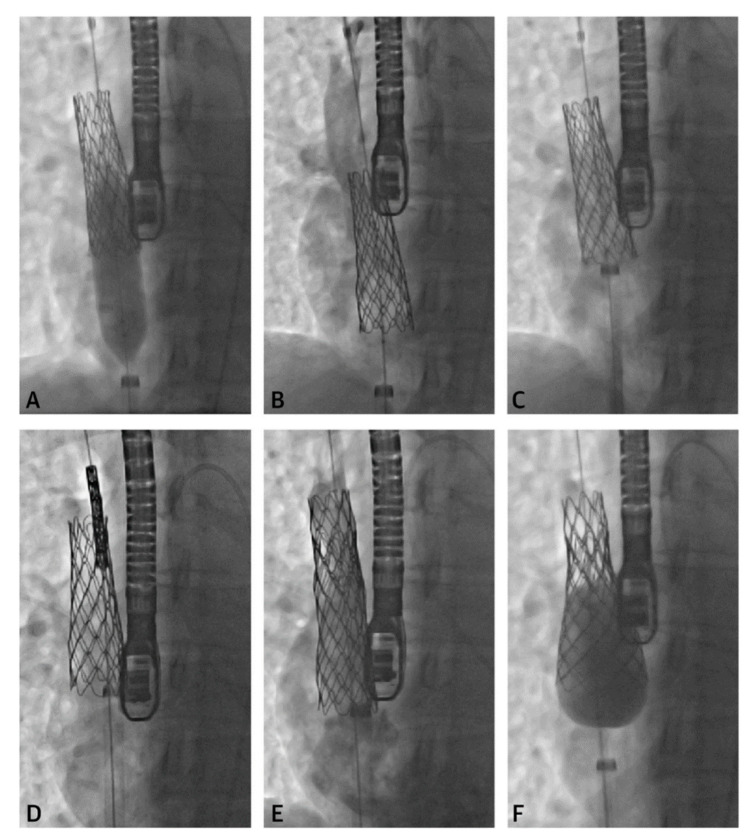
Series of fluoroscopy images depicting stent migration following attempted post-dilation (**A**,**B**). The stent was repositioned with the long sheath (**C**) followed by another stent placement (**D**,**E**). A larger balloon was used to post-dilate the first stent into place (**F**). Revised from ref [4].

**Figure 6:**
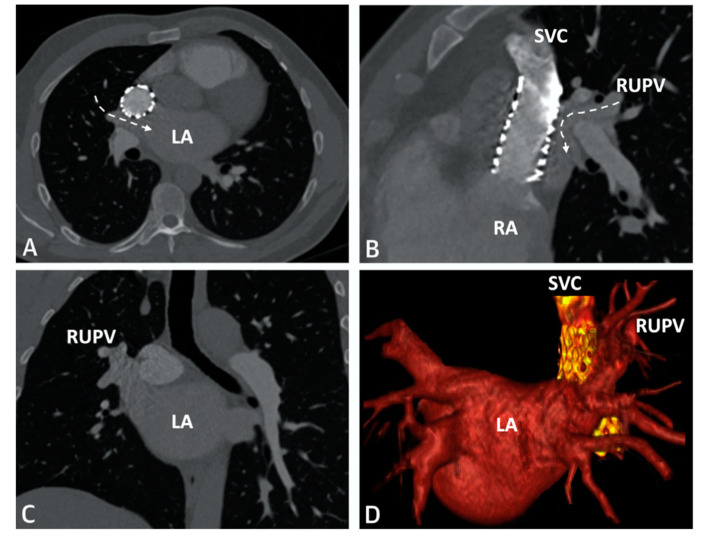
CT imaging following successful covered stent placement demonstrates no pulmonary venous obstruction to the LA (**A**–**C**). A 3D virtual model confirms unobstructed drainage from the pulmonary veins to the LA (**D**). Revised from ref [4].

**Figure 7:**
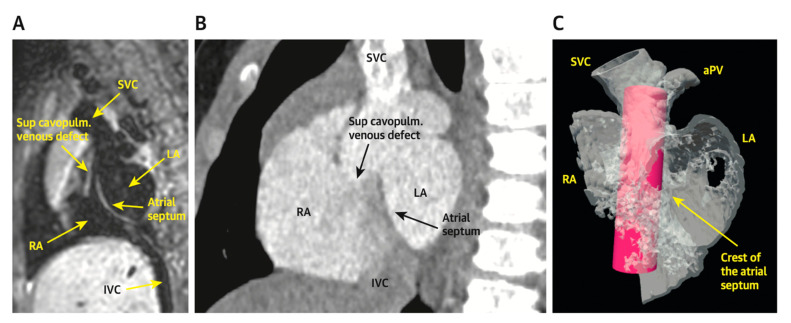
Virtual reality simulation to better understand the relationship of the proposed covered stent to surrounding structures. Both patients in (**A**) (CMR) and (**B**) (CT) were deemed inappropriate candidates for covered stent placement due to SVC angulation to the LA. Imaging from the patient in (**B**) was further confirmed using virtual reality (**C**). The covered stent is represented by the pink cylinder. Revised from ref [5].

The following Figure 1, Figure 2, Figure 3, Figure 4, Figure 5 and Figure 6 describe in detail the pre-procedural, intra-procedural, and post-procedural considerations [4]. Figure 7 demonstrates how the “virtual reality” environment can provide a critical look at the cross-sectional imaging in an interactive platform to deliver a reliable understanding of the patient’s anatomical limitations [5].

### 2.2. Patent Ductus Arteriosus (PDA) Stenting for Ductal-Dependent Pulmonary Blood Flow

PDA stenting is emerging as an alternative to surgical shunts for PDA-dependent pulmonary blood flow in newborns with CHD. Every PDA is not created equal, and there are many considerations that must be evaluated before undertaking PDA stenting.

#### 2.2.1. Pre-Procedural

**Figure 8:**
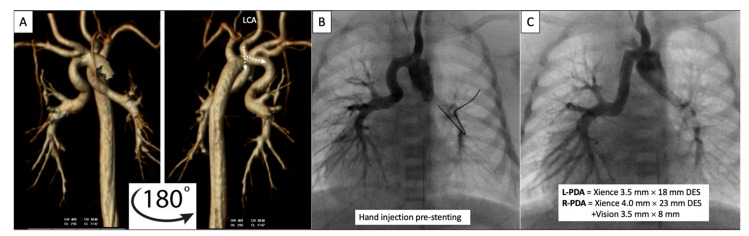
Pre-procedural 3D virtual CT imaging (**A**) predicts the left carotid artery (LCA) as the best access site for bilateral PDA stenting (**B**,**C**) in a patient with discontinuous PAs. Multi-Link Vision and Xience Alpine^TM^ drug eluting (DES) stents (Abbott Vascular Company, Abbott Park, IL, USA) were used. Revised from ref [9].

**Figure 9:**
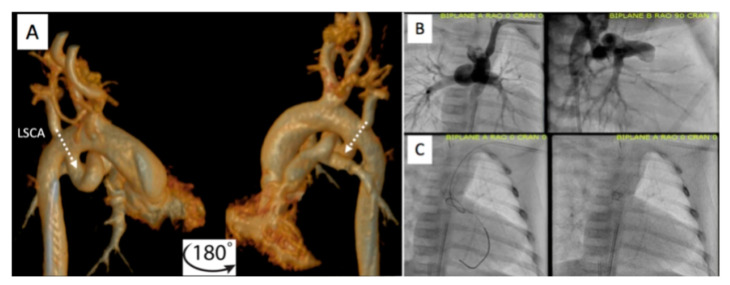
Additional example of pre-procedural CT imaging (**A**) aiding with access site selection for successful PDA stenting (**B**,**C**) via the left axillary artery (LAA) to the left subclavian artery (LSCA). Revised from ref [9].

Prior to catheterization, many centers routinely obtain cross-sectional imaging with contrast CT scan to better assess the take-off, tortuosity, and size of the PDA (Figure 8 and Figure 9) [6,7,8]. A single-center study has shown a statistically significant reduction in number of access sites, contrast exposure, as well as fluoroscopic and procedural time without significantly increasing the cumulative radiation burden.

#### 2.2.2. Post-Procedural

**Figure 10:**
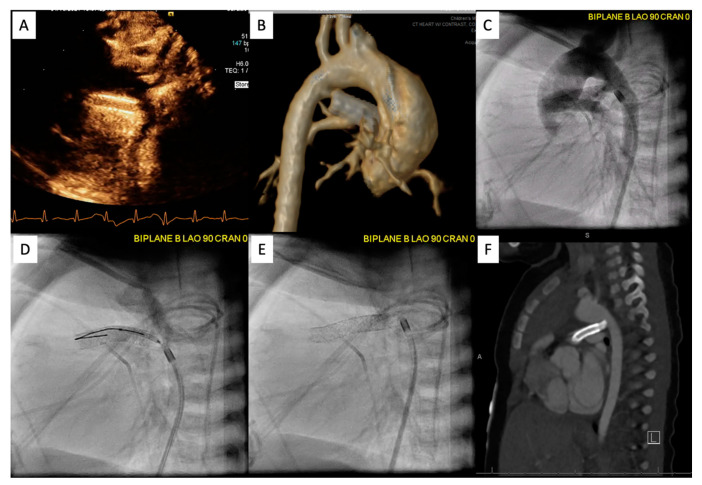
Patient is status post PDA stenting with concern for cyanosis. ECHO/CT scan demonstrated that the aortic end was uncovered (**A**,**B**). Patient returned to the catheterization lab for an additional stent placement (**C**–**E**). Follow up PDA stenting showed full coverage (**F**).

Following the procedure, it is important to continue to monitor saturations to ensure the patient maintains adequate pulmonary blood flow. Figure 10 demonstrates an example of PDA stenting where the aortic end was not fully covered. The patient returned to the catheterization lab after this lesion was identified by CT scan and the stenotic aortic end was fully stented. The patient did well afterwards, saturations stabilized, and the patient was discharged home with close follow-up.

### 2.3. Percutaneous Pulmonary Valve Placement

Pre-Procedural

**Figure 11:**
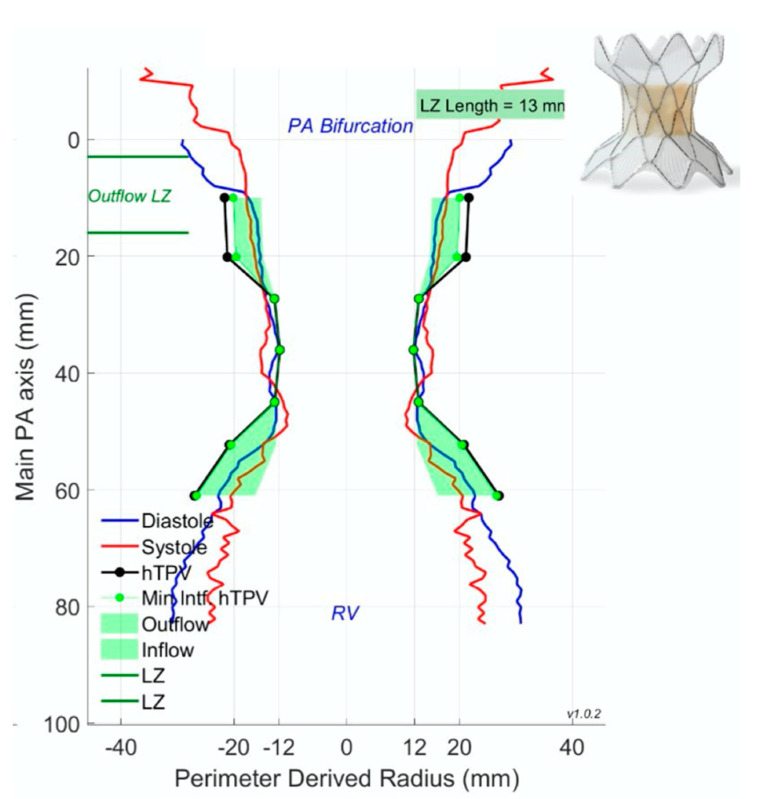
Perimeter plot represents optimal valve placement of the 25 mm Harmony valve (Medtronic, Minneapolis, MN) shown on the top right of the figure. The outflow landing zone (LZ) on the pulmonary artery (PA) end is a landmark for implanters to deliver the distal end of the Harmony valve. Furthermore, blue (diastole) and red (systole) lines depict the variation in cardiac dimensions during cardiac motion for better predictions of valve placement.

**Figure 12:**
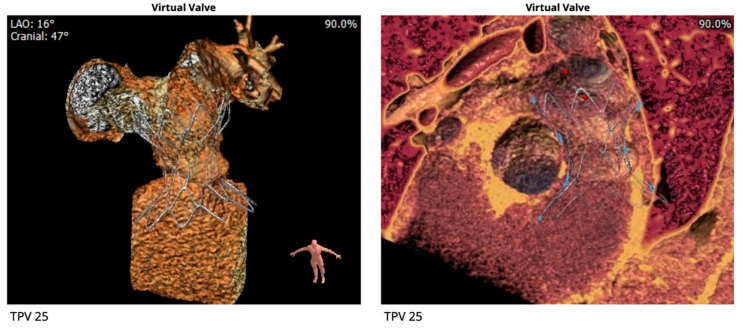
Pre-procedural virtual modeling demonstrates ideal placement of the Harmony valve.

**Figure 13:**
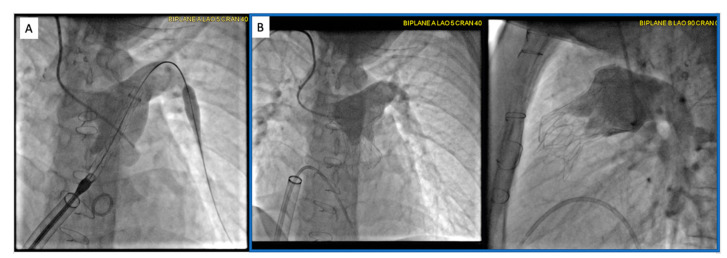
Intra-procedural angiograms demonstrating the 25 mm Harmony valve positioning (**A**) and final placement (**B**) on AP and Lateral projections in a patient with pulsatile Glenn physiology. A 26-French DrySeal sheath was advanced from the femoral venous access to deliver the Harmony valve. Additional access was obtained from the right internal jugular vein for angiography during valve placement.

Cross-sectional imaging is an indispensable tool for patient counseling and planning prior to percutaneous pulmonary valve placement. The three leading Food and Drug Administration (FDA) approved percutaneous pulmonary valves utilized in the United States are (1) the Melody valve (Medtronic, Minneapolis, MN, USA), (2) the Sapien valve (Edwards Lifesciences, Irvine, CA, USA), and (3) the most recently approved Harmony valve (Medtronic, Minneapolis, MN, USA). Pre-procedural cross-sectional imaging allows for improved patient selection prior to catheterization. Patient examples of Harmony valve evaluation and placement are outlined in Figure 11, Figure 12 and Figure 13.

### 2.4. Cross-Sectional Overlay Fusion in the Catheterization Lab

**Figure 14:**
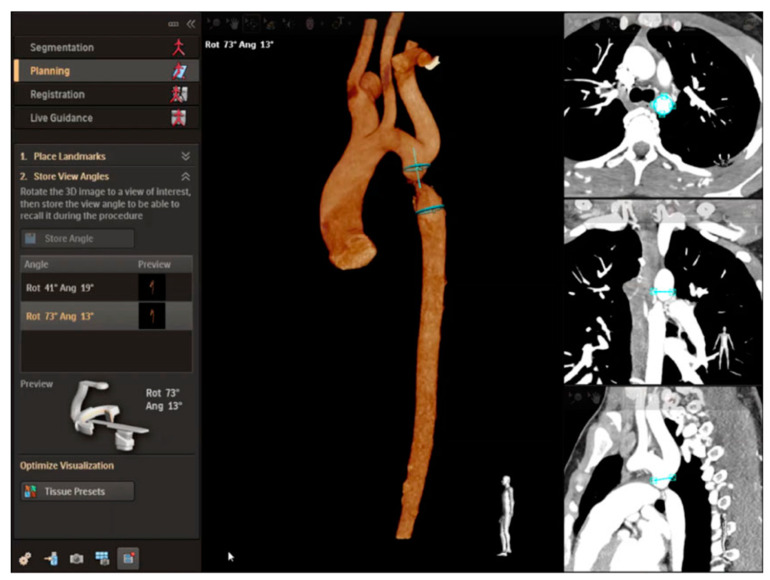
Overlay planning using the VesselNavigator system (Philips Healthcare, Best, The Netherlands) for a patient with a discrete coarctation of the aorta (CoA) prior to stenting using CT images. The area of interest is outlined in blue. Revised from ref [9].

**Figure 15:**
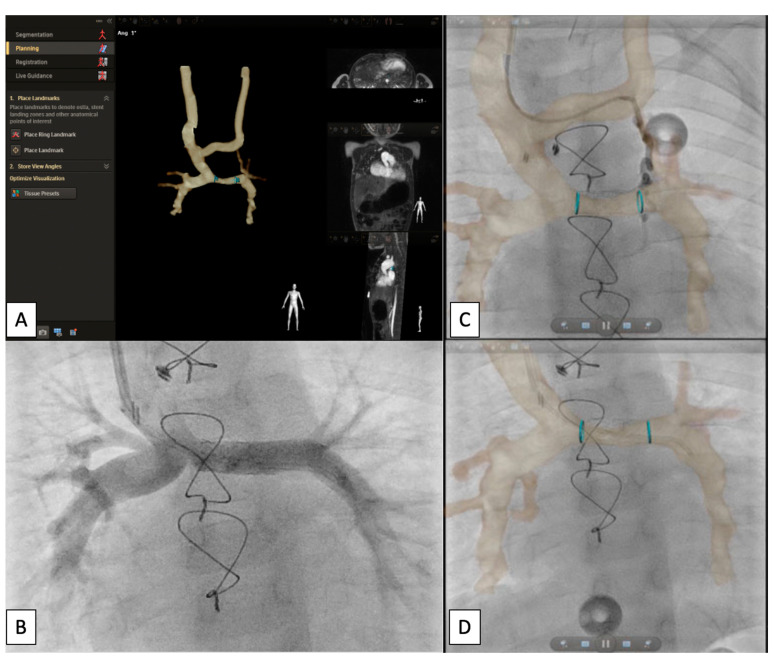
Series of images depicting overlay planning (**A**) and intervention on a small veno-venous collateral (**B**) as well as left pulmonary artery (LPA) stenting (**C**,**D**) in a CHD patient status post Glenn palliation. Revised from ref [9].

**Figure 16:**
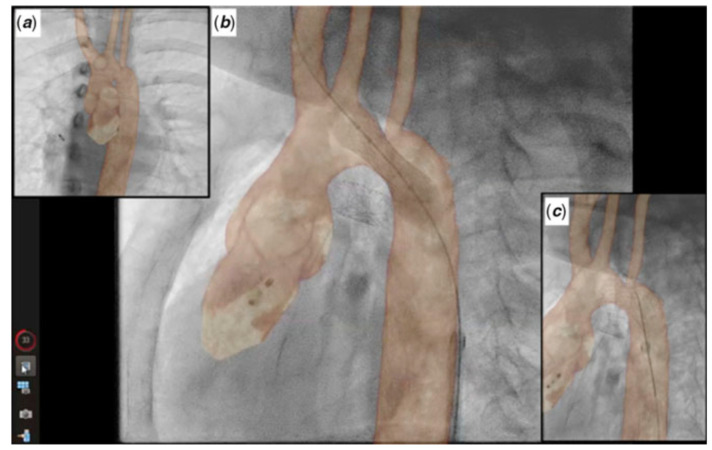
Series of overlay fluoroscopy images depicting CoA stenting at different camera angulations (**a**,**b**) as well as side cell dilation to avoid jailing the LSCA (**c**). Revised from ref [9].

**Figure 17:**
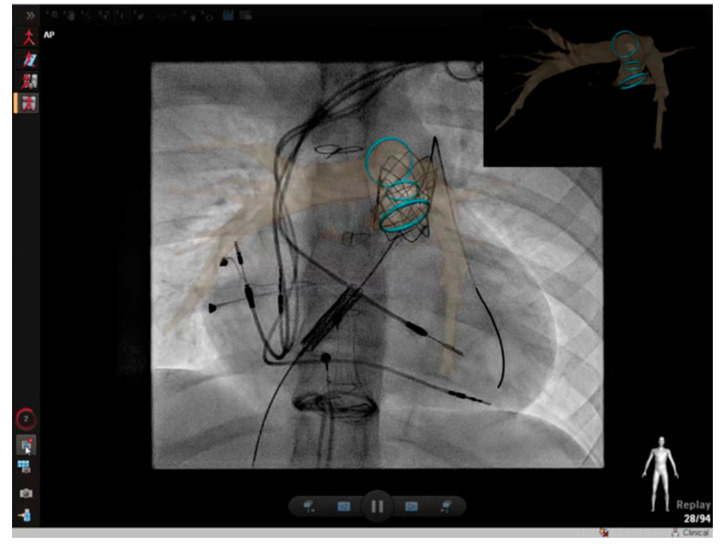
Patient is status post Tetralogy of Fallot conduit repair who now presents for fluoroscopic overlay guided percutaneous stent and pulmonary valve placement. The blue circles represent the optimal landing zone. Revised from ref [9].

**Figure 18:**
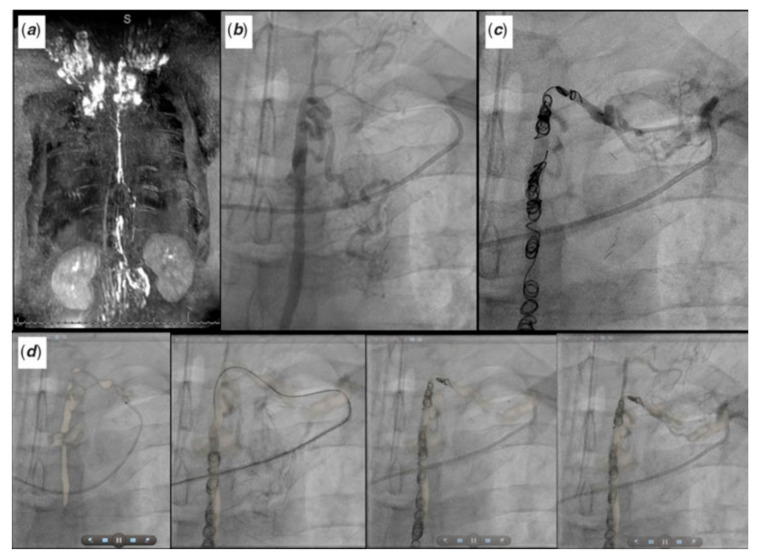
Symptomatic Fontan patient with severe lymphatic insufficiency presents following a dynamic contrast magnetic resonance lymphangiogram (**a**) for fluoroscopic overlay guided retrograde lymphatic embolization (**b**–**d**) via the thoracic duct. Revised from ref [9].

**Figure 19:**
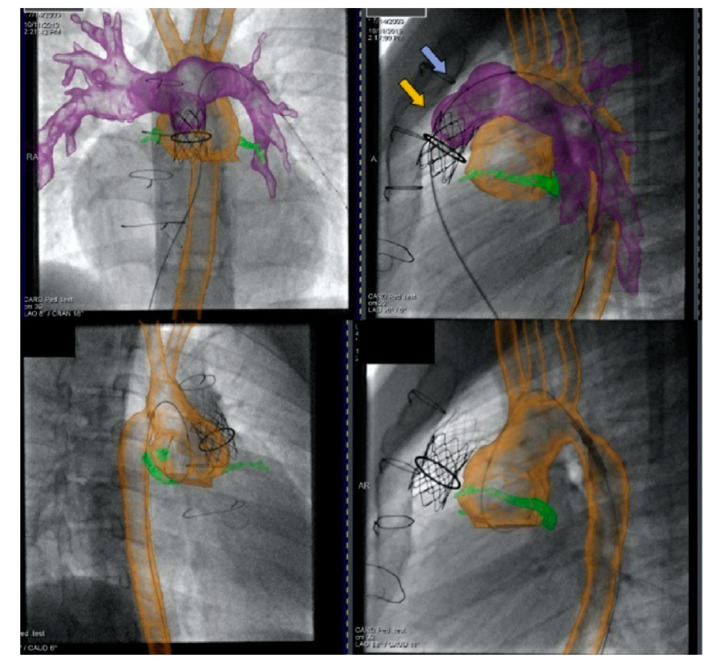
Biplane overlay (Siemens Healthineers, Munich, Germany) to assist with Melody valve placement (top row) followed by coarctation of the aorta (CoA) stenting in the same patient (bottom row). The pulmonary arteries are outlined in purple, and the aorta is outlined in orange. The two arrows point to the bare metal stent (yellow) within the valved conduit and newly placed Melody valve (violet). Ref [13].

Cross-sectional overlay enabled registration of previously acquired CT and/or MRI images is being used to rapidly fuse with X-ray fluoroscopic imaging in single and biplane systems [10,11]. Several single-plane overlay examples (VesselNavigator system (Philips Healthcare, Best, The Netherlands)) are outlined below (Figure 14, Figure 15, Figure 16, Figure 17 and Figure 18) [12]. A biplane overlay example (Siemens bi-plane system (Siemens Healthineers, Munich, Germany) is depicted in Figure 19 [13]. Overlay fusion has demonstrated a reduction in overall radiation burden [8,9].

### 2.5. Coronary Artery Assessment in CHD

**Figure 20:**
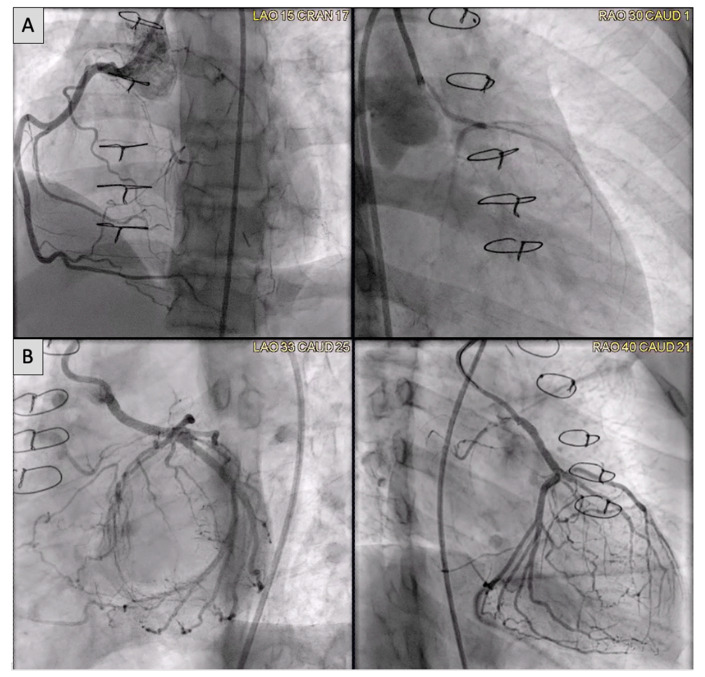
(**A**) Anomalous left coronary artery from the pulmonary artery (ALCAPA) patient status post reimplantation and now found to have severe proximal occlusion of the left main coronary artery (LMCA). (**B**) Post-stent placement in the LMCA with improved antegrade reperfusion on follow up angiography.

**Figure 21:**
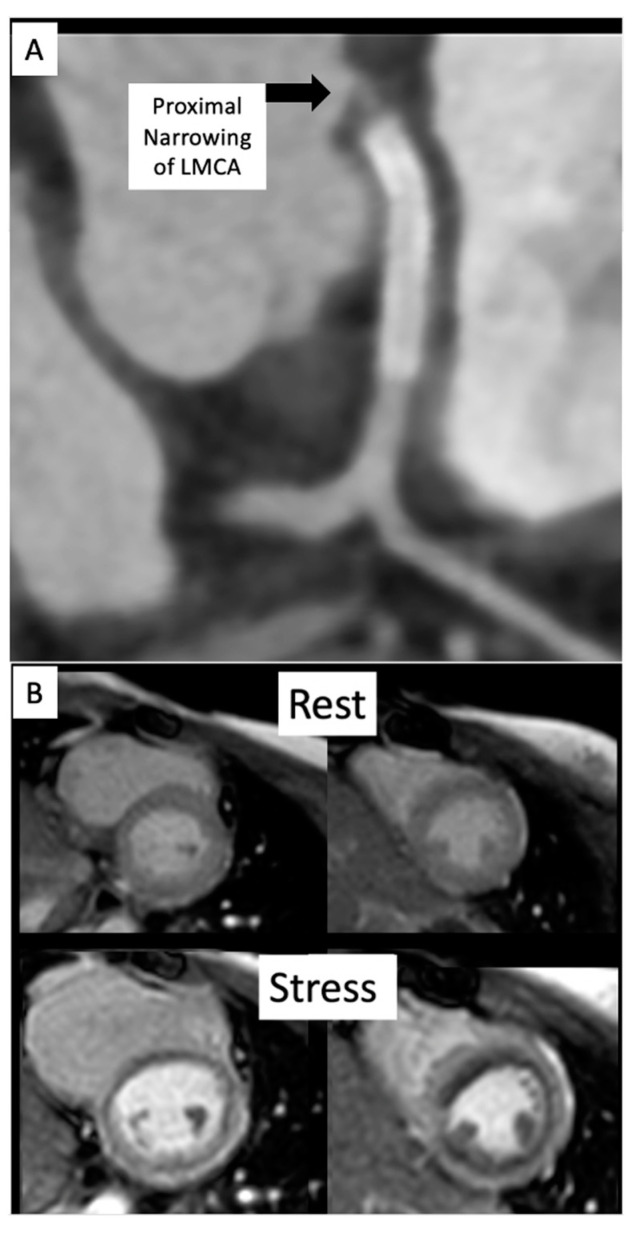
(**A**) CT on follow-up for patient from Figure 20 demonstrates a segment of near complete occlusion at the most proximal aspect of the left main coronary artery (LMCA), immediately prior to the previously placed stent. (**B**) Cardiac MRI demonstrated inducible perfusion defects along the anteroseptal, anterior, anterolateral, and lateral walls, at the basal and mid-ventricular level, as well as in the anterolateral papillary muscle (territory of the LMCA). No areas of late gadolinium enhancement to indicate myocardial fibrosis/injury are present. Patient proceeded to the catheterization lab for additional stenting of the LMCA ostium.

Congenital coronary interventions are rare, and the anatomy for each case is quite varied. Therefore, cross-sectional imaging can be extremely helpful when planning such interventions. An example of an anomalous left coronary artery from the pulmonary artery (ALCAPA) repair with residual left main coronary artery (LMCA) narrowing is outlined in Figure 20 and Figure 21. In addition, ongoing studies aim to assess pediatric coronary allograft vasculopathy with CT angiography and CMR adenosine stress perfusion [14,15] to compliment traditional assessment by X-ray fluoroscopic catheter angiography.

## 3. Future Directions

**Figure 22:**
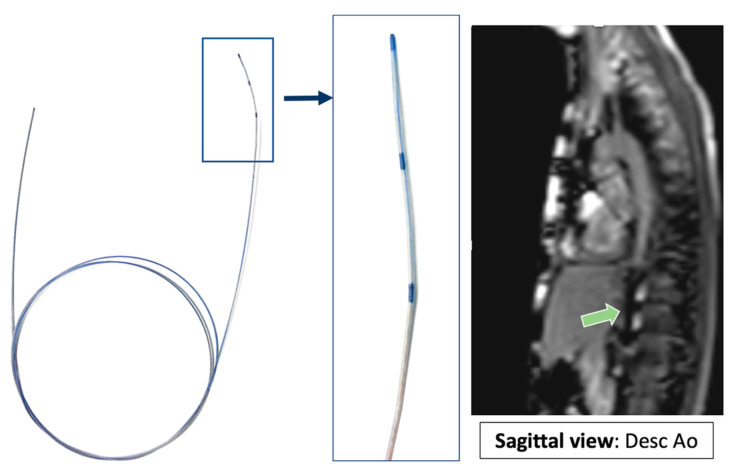
MR guided retrograde left heart catheterization (LHC) with an MR conditional guidewire (EmeryGlide guidewire, Nano4Imaging, Dusseldorf, Germany). Green arrow points to the guidewire artifact produced by the three markers on the tip of the wire. Revised from ref [16].

**Figure 23:**
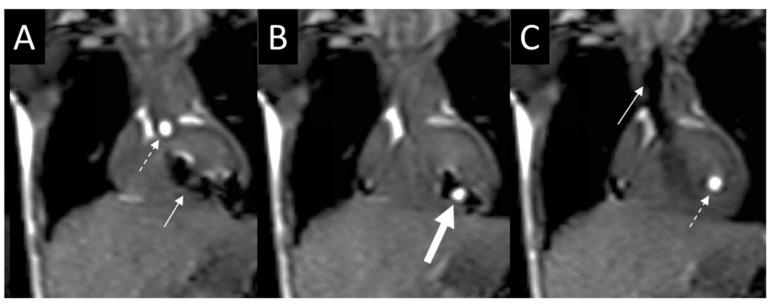
Series of images (**A**–**C**) in the coronal plane demonstrating step by step entrance into the left ventricle (LV) for pressure measurements using a gadolinium-filled balloon-tip catheter (dashed arrow) and the MR conditional guidewire (solid arrow). The thick solid white arrow is pointing to both the catheter and wire together within the LV. Revised from ref [16].

**Figure 24:**
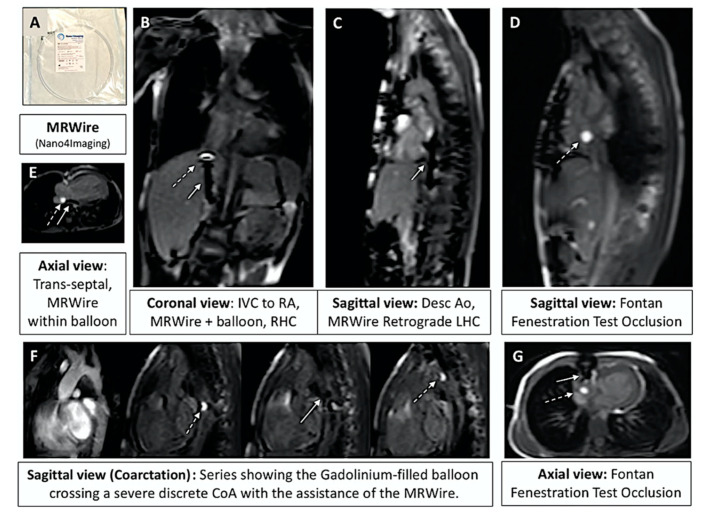
Overview of several examples using iCMR. (**A**) Packaging for the MR-conditional guidewire used to navigate catheters. (**B**) Prograde right heart catheterization (RHC) with the gadolinium-filled balloon-tip catheter (dashed arrow) and the MR conditional guidewire (solid arrow) positioned within IVC just prior to the RA entrance. (**C**) Retrograde LHC with the MR-conditional guidewire within the descending aorta at the level of the diaphragm. (**D**) Sagittal view of a Fontan fenestration test occlusion (FFTO) to test eligibility for device closure. (**E**) Prograde evaluation of pulmonary veins across the atrial septal defect. (**F**) Retrograde LHC in a patient with CoA to define gradient prior to transfer to the fluoroscopy suite for stent placement. (**G**) Additional view from an axial view demonstrating FFTO to test eligibility for device closure. Revised from ref [16].

**Figure 25:**
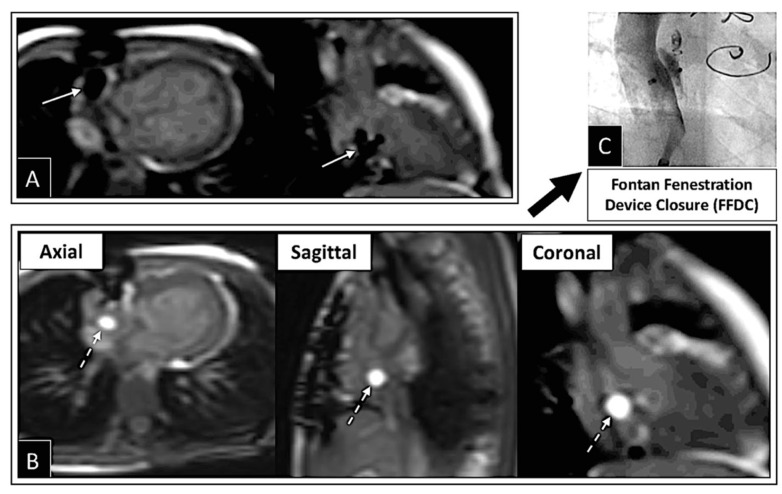
Series of images demonstrating iCMR Fontan fenestration test occlusion (FFTO) leading to device closure in the fluoroscopy lab. (**A**) iCMR guidance as the MR-conditional wire crosses the Fontan fenestration. Wire position is confirmed in multiple planes. (**B**) Multiple planes demonstrating FFTO to test eligibility for device closure. (**C**) Patient was deemed appropriate and transferred to the fluoroscopy X-ray suite where Fontan fenestration device closure (FFDC) was performed. Revised from ref [16].

Invasive CMR (iCMR) is an attempt at incorporating the best of both modalities [16,17,18,19,20]. There is concern that radiation exposure during childhood may predispose this population to increased risk of cancer later in life [21]. By pursuing catheter-based procedures under MR guidance instead of X-ray fluoroscopy, patients will benefit from reduction in overall procedural radiation exposure. iCMR utilizes a dilute gadolinium-filled balloon-tip catheter in combination with an MR-conditional guidewire to obtain cardiac hemodynamics with real-time CMR guidance. Examples of iCMR equipment and procedures are outlined in Figure 22, Figure 23, Figure 24 and Figure 25.

## 4. Discussion

CT and CMR continue to advance the field of congenital cardiology by allowing for new and unique procedures not previously imagined. Enabling the interventionalist to enter the catheterization lab with a plan of attack expedites the procedure and has been shown to reduce procedural times and improve outcomes.

Substantial advances in cross-sectional image resolution with a significant decrease in radiation exposure for cardiac CTs have led to more mainstream utilization. CMR has a unique ability to deliver real-time functional imaging in several views without exposing the patient to the detrimental effects of ionizing radiation. This can reduce procedural times in the interventional fluoroscopic suite to allow for more directed procedures. iCMR evaluations are especially beneficial for the pulmonary hypertension [22] and single-ventricle patient populations as assessment by cardiac catheterization alone may not be adequate to assess important variables such as cardiac flows [23,24] and lymphatic physiology [25].

## 5. Conclusions

A significant shift toward pre-procedural planning utilizing cross-sectional imaging and 3D reconstruction is now being embraced by congenital heart centers to better serve their patients. With continual advancements, we anticipate new interventions and improved outcomes for the future of congenital cardiology.

## Data Availability

Not applicable.

## Abbreviations

Congenital heart disease	CHD
Computed tomography	CT
Cardiac magnetic resonance imaging	CMR
Sinus venosus atrial septal defect	SVASD
Superior vena cava	SVC
Right upper pulmonary vein	RUPV
Left atrium	LA
Right atrium	RA
3 dimensional	3D
Aorta	Ao
Right middle pulmonary vein	RMPV
Anomalous pulmonary vein	aPV
Inferior vena cava	IVC
Patent ductus arteriosus	PDA
Drug eluting stent	DES
Left carotid artery	LCA
Left axillary artery	LAA
Left subclavian artery	LSCA
Food and drug administration	FDA
Landing zone	LZ
Pulmonary artery	PA
Left pulmonary artery	LPA
Coarctation of the aorta	CoA
Anomalous left coronary artery from the pulmonary artery	ALCAPA
Left main coronary artery	LMCA
Invasive CMR	iCMR
Left ventricle	LV
Right heart catheterization	RHC
Left heart catheterization	LHC
Fontan Fenestration Test Occlusion	FFTO
Fontan fenestration device closure	FFDC
